# Focal Thyroid Incidentalomas on PET/CT Among Breast Cancer Patients: Referral Patterns and Diagnostic Outcomes

**DOI:** 10.3390/diagnostics16142231

**Published:** 2026-07-16

**Authors:** Majd Asakly, Adi Sharabi-Nov, Moran Barazani-Avitan, Jamal Gantus, Ahmad Khalaila, Haia Darawshi, Rabie Shehadeh, Asaf Bin Simon, Yaniv Avraham, Michael Edelstein, Moshe Bocher, Israel Sandler, Fauzi Artul, Aviva Ron, Shlomo Merchavy

**Affiliations:** 1 Otolaryngology, Head & Neck Surgery Unit, Ziv Medical Center, Safed 13100, Israelmerchavy@gmail.com (S.M.); 2 Azrieli Faculty of Medicine, Bar-Ilan University, Safed 1311502, Israel; 3 Research Department, Ziv Medical Center, Safed 13100, Israel; 4 Tel-Hai University of Kiryat Shmona, Shmona 11016, Israel; 5 Nuclear Medicine Department, Ziv Medical Center, Safed 13100, Israel

**Keywords:** thyroid incidentaloma, focal thyroid uptake, incidental thyroid carcinoma, breast cancer, ^18^F-FDG PET/CT, positron emission tomography/computed tomography, thyroid malignancy, papillary thyroid carcinoma, standardized uptake value (SUVmax), fine-needle aspiration

## Abstract

**Background**: The increasing use of fluorine-18 fluorodeoxyglucose positron emission tomography/computed tomography (^18^F-FDG PET/CT) in patients with breast cancer has led to a growing number of incidentally detected thyroid lesions. Thyroid incidentaloma refers to a focal area of increased metabolic activity within the thyroid gland. Up to 35% of focal thyroid incidentalomas are malignant. Early detection of thyroid carcinoma may allow less invasive surgical management and reduce the need for more extensive treatment associated with advanced disease, including total thyroidectomy, neck dissection, and radioactive iodine therapy. The objective of this study was to estimate the prevalence of focal thyroid incidentaloma and incidental thyroid carcinoma detected on PET/CT scans performed in breast cancer patients at a single tertiary medical center serving a highly ethnically diverse population. **Methods**: Medical records and PET/CT scans were retrospectively reviewed at a single medical center. Data collected included the presence of thyroid incidentaloma, maximum standardized uptake value (SUVmax), thyroid cytology, primary malignancy type, breast cancer status, ethnicity, and age. A total of 1233 patients with cancer who underwent PET/CT imaging and received treatment at ZIV Medical Center between August 2018 and December 2024 were included. Forty-two patients with primary thyroid carcinoma or head and neck carcinoma were excluded. Patients were categorized into breast cancer and non-breast cancer groups and compared regarding the prevalence of thyroid incidentaloma, referral rates for further evaluation by head and neck specialists, and the rate of incidental thyroid carcinoma. **Results**: Among 330 patients with breast cancer, 16 (4.8%) had a focal incidental thyroid finding, compared with 24 (2.8%) of 861 patients with non-breast malignancies who underwent PET/CT imaging. Among the 16 breast cancer patients with thyroid incidentaloma, thyroid carcinoma was subsequently confirmed in 4 patients (25%) who underwent further evaluation. The proportion of patients with confirmed thyroid carcinoma among those with incidentalomas was 1.2% (4/330) in the breast cancer group compared with 0.46% (4/861) in the non-breast cancer group. Approximately half of the patients in both groups were referred for further thyroid evaluation. Among breast cancer patients, non-significant trends toward higher referral rates were observed in patients with non-advanced disease, higher SUVs, and Jewish ethnicity. Nearly all patients evaluated by head and neck specialists underwent fine-needle aspiration (FNA). The mean SUV among patients diagnosed with papillary thyroid carcinoma (PTC) on FNA (*n* = 8; breast and non-breast cancer combined) was 15.2 (IQR: 7.3–17.0), compared with 5.2 (IQR: 5.0–6.0) among patients with benign cytology (*n* = 5; *p* = 0.011). **Conclusions**: Thyroid incidentalomas identified on PET/CT scans in breast cancer patients were relatively common in this cohort, and a proportion of evaluated lesions were subsequently diagnosed as thyroid carcinoma. Referral and diagnostic follow-up rates were variable, with approximately half of patients referred for further evaluation and only one-third attending a head and neck clinic. Given the retrospective design, limited number of biopsy-confirmed cases, and potential verification bias, these findings should be interpreted with caution. Nevertheless, they highlight the importance of clinical awareness and support individualized assessment of PET/CT-detected thyroid incidentalomas rather than broad management recommendations.

## 1. Introduction

The expanding use of fluorine-18 fluorodeoxyglucose positron emission tomography/computed tomography ^18^F-FDG PET/CT) in patients with breast cancer has led to the increasing detection of incidental focal uptake within the thyroid gland [1,2]. These focal uptakes are referred to as thyroid incidentalomas. Thyroid incidentalomas are identified on imaging studies performed in patients without thyroid-related symptoms, clinical findings, or prior suspicion of thyroid disease [3]. The reported prevalence of focal thyroid incidentaloma on PET/CT ranges from 1% to 3% [2,3]. However, this prevalence increases to as high as 14% when diffuse and mixed uptake patterns are included [4]. While diffuse uptake is more commonly associated with inflammatory conditions such as thyroiditis, focal uptake may indicate underlying malignancy [5].

Patients with breast cancer undergo PET/CT imaging as part of disease staging, assessment of disease burden, and evaluation of treatment response. The risk of developing a second primary thyroid malignancy is increased among breast cancer patients. The standardized incidence ratio for developing thyroid cancer following breast cancer has been reported as 1.73 [6]. A meta-analysis demonstrated an odds ratio of 1.55 for the development of thyroid cancer as a secondary malignancy after breast cancer diagnosis and treatment [7]. Similarly, Liu et al. reported an increased risk of thyroid cancer among breast cancer patients (OR = 1.17, *p* = 0.018) [8]. Patients diagnosed with both breast and thyroid cancer have been described as sharing a characteristic profile including younger age, estrogen receptor positivity, and familial predisposition [9].

Focal thyroid incidentalomas may harbor malignancy in 25–50% of cancer patients [5,10,11,12]. It is noteworthy that thyroid cancer occurring in patients with breast cancer has been associated with more favorable outcomes compared with thyroid cancer in patients without breast cancer [13]. In addition, increased standardized uptake value (SUV) of thyroid incidentalomas may be associated with a higher likelihood of malignancy [14,15,16].

In the present study, the SUV, cytological findings, histopathological findings, and ultrasound (US) characteristics of all thyroid incidentalomas identified on PET/CT scans at a regional oncology center over a five-year period were evaluated. The primary objective of this study was to determine the prevalence of thyroid incidentalomas and incidental thyroid carcinoma among breast cancer patients. The secondary objective was to compare clinical characteristics between patients who were referred for further evaluation and those who were not, and to assess differences in SUVs between malignant and benign thyroid incidentalomas.

## 2. Materials and Methods

A retrospective cohort study was conducted at Ziv Medical Center, a single referral center in Safed, Israel. The study protocol was approved by the Institutional Review Board (IRB) of Ziv Medical Center (Protocol No. 0066-21-ZIV). Hospital records, PET/CT scans, and imaging reports of 1233 patients with cancer who underwent ^18^F-FDG PET/CT imaging in the Nuclear Medicine Department between August 2018 and December 2024 were retrospectively reviewed to identify thyroid incidentalomas.

All patients were at different stages of oncologic care, including initial diagnosis, active treatment, and post-treatment follow-up. Only patients with a single documented primary malignancy were included. Patients with known head and neck malignancies or hematological malignancies were excluded to minimize potential confounding factors. Patients with primary thyroid cancer or other primary head and neck malignancies were also excluded, as these patients routinely undergo comprehensive head and neck evaluation, including dedicated thyroid assessment, as part of their diagnostic workup.

All PET/CT reports were stored in an electronic Radiology Information System (RIS) database. A systematic manual and keyword-based search was conducted across all reports to identify cases documenting thyroid incidentalomas. All PET/CT scans were independently reviewed by the primary author; findings were subsequently re-evaluated by a consultant nuclear radiologist, and a final determination regarding the presence of a thyroid incidentaloma was made.

SUVmax values within thyroid lesions were extracted from PET/CT reports. Only lesions demonstrating focal thyroid uptake were included in this study. Diffuse thyroid uptake, typically associated with inflammatory conditions such as thyroiditis and not commonly linked to an increased risk of malignancy, was excluded and was not considered an incidentaloma.

**Figure 1:**
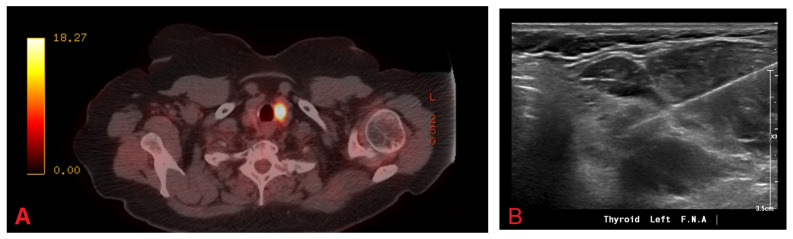
(**A**) Focal incidentaloma in the left thyroid lobe on fluorine-18 fluorodeoxyglucose positron emission tomography/computed tomography (^18^F-FDG PET/CT), with a maximum standardized uptake value (SUVmax) of 18. (**B**) Ultrasound-guided fine-needle aspiration (FNA). Cytology demonstrated papillary thyroid carcinoma. The final diagnosis was confirmed on postoperative histopathological examination following left hemithyroidectomy.

SUV max values of thyroid lesions were extracted from PET/CT reports. Only lesions demonstrating focal thyroid uptake were included in the study. Diffuse thyroid uptake, which is typically associated with inflammatory conditions such as thyroiditis and is not commonly linked to an increased risk of malignancy, was excluded and was not considered a thyroid incidentaloma. For the purposes of this study, a focal thyroid incidentaloma was defined as a localized area of increased metabolic activity within the thyroid gland. This could involve part of a single thyroid lobe or both lobes, and the definition was not restricted to well-demarcated lesions (Figure 1). Areas of focal uptake with ill-defined margins were also included, as clinically significant malignancies may occur in such findings and may not always present as sharply demarcated foci on PET/CT imaging.

When SUVmax values were not available in the original PET/CT report, the scans were re-evaluated and SUVmax was calculated by a consultant radiologist. Subsequently, electronic medical records were reviewed to identify additional thyroid-related investigations and management, including thyroid ultrasound, fine-needle aspiration (FNA) cytology, and surgical intervention. The review was extended to paper-based oncology records, as some investigations may have been performed outside the institution and were therefore not available in the electronic medical record system.

Collected clinical data included age, sex, ethnicity, primary disease status, SUVmax of thyroid incidentalomas, and cytological findings obtained from fine-needle aspiration (FNA) of thyroid nodules. Disease status was classified as advanced (metastatic disease or persistent disease despite oncologic therapy, associated with poor prognosis or substantial morbidity) or non-advanced (stable disease or presumed remission with a favorable prognosis). Patients with known or newly diagnosed thyroid neoplasia or other head and neck malignancies were excluded.

All PET/CT examinations were performed using a dedicated PET/CT scanner (Siemens Biograph LSO, Erlangen, Germany). PET imaging was acquired from the vertex to the mid-thigh using seven bed positions with an acquisition time of 3 min per position. For attenuation correction and anatomical correlation, a low-dose non-contrast CT scan was performed (single-slice, 5 mm slice thickness, 110 mAs, 130 kV). Patients fasted for at least 6 h before FDG administration and had blood glucose levels below 180 mg/dL (10 mmol/L). The administered FDG dose ranged from 333 to 444 MBq (9–12 mCi). No adjustments to SUVmax values were made based on blood glucose levels or recovery coefficients. PET/CT images were reviewed in the coronal, axial, and sagittal planes.

All imaging data were reviewed for the purposes of this study. Updated interpretations were communicated to the referring oncologists. During the study period, neither patients nor referring physicians were directly contacted. Decisions regarding further evaluation, including cytological assessment, were made at the discretion of the treating oncologists and primary care physicians, based on clinical judgment and disease stage. Correlations between cytological findings, SUVmax values, age, ethnicity, and primary disease status were subsequently analyzed.

### Statistical Analysis

Differences in age and SUVmax between the study groups were analyzed using the Mann–Whitney U test. Pearson’s chi-square test or Fisher’s exact test was used, as appropriate, to evaluate the associations between the study groups and categorical variables (gender, ethnicity, and advanced disease). A two-sided *p*-value < 0.05 was considered statistically significant. Statistical analyses were performed using SPSS version 29 (IBM).

## 3. Results

**Figure 2:**
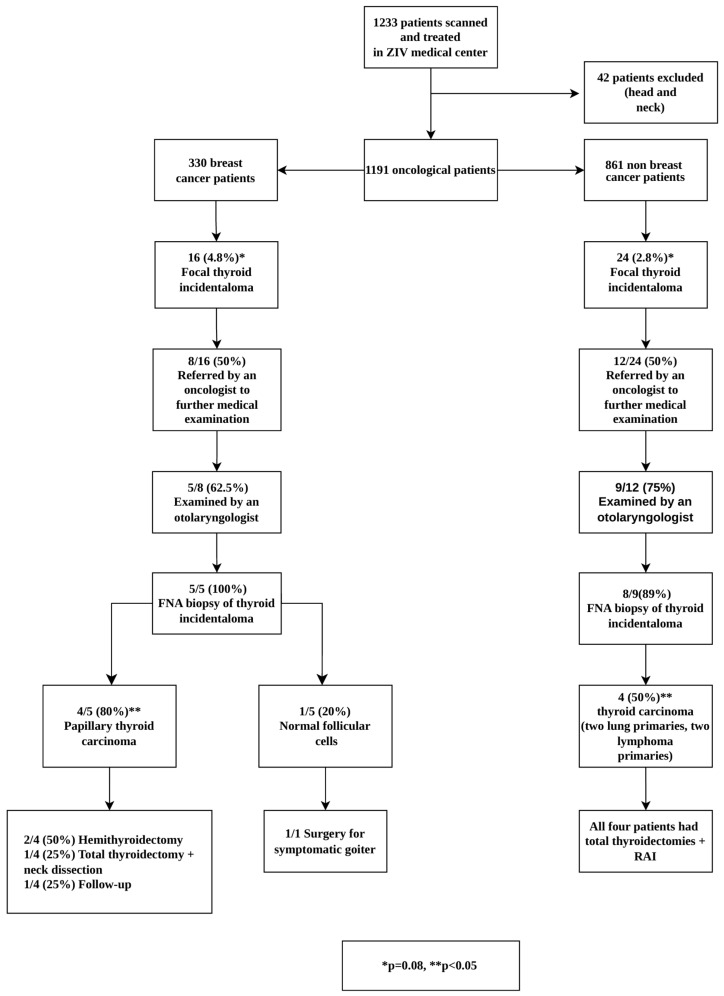
Patient flowchart.

PET/CT scans of 1233 patients treated at Ziv Medical Center between August 2018 and December 2024 were reviewed (Figure 2). Forty-two patients with primary thyroid carcinoma or head and neck malignancies were excluded. The overall rate of thyroid incidentaloma in the study cohort was 3% (40/1191).

Among 330 patients with breast cancer, 16 (5%) had a focal incidental thyroid finding, compared with 24 (3%) of 861 patients in the non-breast cancer group. This difference did not reach statistical significance (*p* = 0.08).

In both groups, only approximately half of the patients with thyroid incidentalomas were referred for further evaluation by the treating oncologist. Non-significant numerically higher referral rates were observed in patients with non-advanced disease, higher SUVmax values, and Jewish ethnicity (Table 1). However, these trends did not reach statistical significance.

Among patients referred to a head and neck specialist, approximately two-thirds underwent further clinical evaluation. Nearly all patients assessed by a head and neck specialist underwent fine-needle aspiration (FNA), except for one case.

Papillary thyroid carcinoma (PTC) was confirmed in 8 of 40 patients with thyroid incidentalomas. Among biopsied lesions, at least 62% (8/13) were malignant: 80% (4/5) in the breast cancer group and 50% (4/8) in the non-breast cancer group (*p* < 0.05). These biopsy-based findings should be interpreted cautiously, as only a selected subset underwent specialist assessment and FNA. In the non-breast cancer group, these patients had underlying lung cancer or lymphoma.

Among patients with thyroid incidentalomas, thyroid carcinoma was identified in 25% (4/16) of the breast cancer group and 17% (4/24) of the non-breast cancer group. Overall, confirmed thyroid carcinoma was identified in 1.2% (4/330) of the breast cancer cohort and 0.46% (4/861) of the non-breast cancer cohort (*p* = 0.17).

All patients diagnosed with PTC underwent surgical treatment, including partial or total thyroidectomy, with or without neck dissection, and with or without adjuvant radioactive iodine therapy (Table 2). Surgery was not performed in one case involving an 84-year-old patient with metastatic breast cancer due to advanced systemic disease.

The mean SUVmax in patients with PTC diagnosed on FNA (*n* = 8; breast and non-breast cancer combined) was 15.2 (IQR: 7.3–17.0), compared with 5.2 (IQR: 5.0–6.0) in patients with benign cytology (*n* = 5; one breast cancer and four non-breast cancer patients), with a statistically significant difference (*p* = 0.011).

## 4. Discussion

### 4.1. Prevalence of Thyroid Incidentaloma

PET/CT is an essential imaging modality for the assessment and follow-up of patients with breast and other malignancies. It is widely used for staging, evaluation of tumor burden, detection of metastases, and assessment of treatment response. Each year, a large number of such examinations are performed in Israel.

The reported prevalence of focal thyroid incidentalomas on PET/CT ranges from 1% to 3% [3,12]. In the present study, the overall prevalence of focal thyroid incidentaloma was 3% (40/1191), including 5% (16/330) in breast cancer patients and 3% (24/861) in non-breast cancer patients, which is consistent with previously published data. The variability in reported prevalence across studies may be attributed to the lack of a universally accepted definition of thyroid incidentaloma, differences in PET/CT acquisition protocols between centers, variability in radiological interpretation, and potential medico-legal considerations influencing reporting practices.

Interpretation of focal thyroid uptake may also vary among physicians, with some considering only well-demarcated foci as incidentalomas, while others include less clearly defined uptake patterns. In this study, a broad definition of focal thyroid incidentaloma was applied, including any focal metabolic activity within the thyroid gland, even in the absence of sharp demarcation. This is a universally accepted strict definition in the literature. Accordingly, lesions with low-grade metabolic activity are included based on evidence suggesting that even these findings may harbor malignancy.

### 4.2. Prevalence of Malignancy in Thyroid Incidentaloma

According to Bertagna et al., up to 35% of focal thyroid incidentalomas may be malignant [1]. In previous reports, malignancy rates of 50% were described in selected cohorts (Kao and Yung et al. [11,12]). In this study, the overall prevalence of thyroid carcinoma among thyroid incidentalomas was 20% (8/40).

However, this estimate reflects only patients who underwent further evaluation and FNA. Since only 13 of 40 incidentalomas were biopsied, the observed malignancy rate among biopsied lesions (8/13, 62%) represents a selected subgroup with higher clinical or imaging suspicion. Therefore, this proportion should not be interpreted as the true prevalence of malignancy among all incidental thyroid findings in the cohort.

This selection bias is likely influenced by clinical judgment, imaging characteristics, SUVs, patient prognosis, and referral behavior. Consequently, the true prevalence of malignancy among all PET/CT-detected thyroid incidentalomas in our population remains uncertain.

Despite this limitation, the findings support the notion that clinically significant thyroid malignancies may be detected among PET/CT incidentalomas, underscoring the importance of careful evaluation rather than dismissing these findings as benign.

Only a subset of patients with focal thyroid incidentalomas is referred for further investigation [10,12]. This may reflect the absence of a standardized management pathway once such findings are identified on PET/CT. In our cohort, only 20 of 40 patients with thyroid incidentalomas were referred for further evaluation by a head and neck specialist. Of those referred, approximately two-thirds attended the clinic, and nearly all underwent FNA, with the exception of one patient. Previous studies implementing structured evaluation protocols have reported investigation rates of up to 66% [17].

Current guidelines from the American Thyroid Association (ATA), European Thyroid Association (ETA), and British Thyroid Association (BTA) recommend ultrasound-guided FNA for focal thyroid uptake detected on ^18^F-FDG PET/CT. Nevertheless, this study demonstrates a relatively low investigation rate [18,19].

Although no statistically significant associations were identified, numerically higher referral rates were observed among patients with non-advanced primary disease, higher SUVs, and Jewish ethnicity. Given the limited sample size and descriptive nature of the analysis, these observations should be considered exploratory and hypothesis-generating and should not be interpreted as evidence of independent predictors of referral (Table 1).

The evaluation of incidental findings that may not impact overall prognosis in patients with advanced malignancy is often clinically complex. The American College of Radiology (ACR) recommends FNA for PET/CT-detected thyroid incidentalomas, except in patients with limited life expectancy [20]. In our cohort, approximately one-quarter of patients died within one year of imaging and may not have been suitable candidates for further invasive evaluation. In other cases, diagnostic workup may have been deferred due to prioritization of active oncologic treatment. Nevertheless, given the increasing use of PET/CT across a broad range of indications, standardized guidelines for incidental findings remain essential.

### 4.3. Correlation Between Thyroid and Breast Cancer

Among biopsied lesions, at least 62% (8/13) were malignant: 80% (4/5) in breast cancer patients and 50% (4/8) in non-breast cancer patients who underwent FNA (*p* < 0.05). The prevalence of thyroid carcinoma in the breast cancer group was 1.2% (4/330), compared with 0.46% (4/861) in the non-breast cancer group (*p* = 0.17).

The prevalence of thyroid carcinoma among incidentalomas in breast cancer patients was higher than in non-breast cancer patients (25% vs. 17%, *p* < 0.05).

In a large cohort, the observed-to-expected (O/E) ratio for thyroid carcinoma in breast cancer patients was 3.7 (*p* < 0.01) [21]. In patients treated with radiotherapy, the O/E ratio increased to 5.5, although this was not statistically significant. An et al. reported a standardized incidence ratio of 2.2 for secondary thyroid carcinoma following breast cancer (*p* < 0.01) [6]. In a study by Nielsen et al. [7], an increased risk of breast cancer as a secondary malignancy following thyroid cancer was observed (OR = 1.55; 95% CI, 1.44–1.67), as well as an increased risk of thyroid cancer as a secondary malignancy following breast cancer (OR = 1.18; 95% CI, 1.09–1.26). Furthermore, using Mendelian randomization analysis, Liu et al. demonstrated a genetic susceptibility to breast cancer that was strongly correlated with an increased risk of thyroid cancer [8].

Hormonal mechanisms may contribute to this association [22]. Thyroid tumor tissue may express estrogen receptors, suggesting a role for estrogen signaling in thyroid carcinogenesis [23]. Estrogen receptor-mediated pathways may enhance the proliferation, migration, and invasiveness of thyroid cancer cells [24].

In Shuhuang Lin’s study, patients with follicular or papillary thyroid carcinoma and a history of breast cancer were compared with patients who had thyroid cancer alone. The authors analyzed data from 604 patients with follicular thyroid carcinoma and 5598 patients with papillary thyroid carcinoma. Interestingly, a history of breast cancer was associated with a lower incidence of distant metastases in both papillary (2.4% vs. 3.0%) and follicular (6.1% vs. 9.1%) thyroid carcinoma (*p* < 0.05). Furthermore, a history of breast cancer was associated with improved survival outcomes, with hazard ratios for mortality of 0.472 (95% CI, 0.370–0.601) for papillary thyroid carcinoma and 0.656 (95% CI, 0.461–0.934) for follicular thyroid carcinoma [13]. These findings are particularly noteworthy, as they further support the complex and intriguing relationship between breast cancer and thyroid cancer and highlight the need for additional research to better understand the biological and clinical mechanisms underlying this association.

Given the favorable prognosis of breast cancer patients, appropriate evaluation and referral of incidental thyroid findings are warranted.

### 4.4. SUV

In the biopsied subgroup, higher SUVs were associated with papillary thyroid carcinoma; however, verification bias and small sample size limit generalizability.

Previous studies have reported conflicting findings regarding SUV as a predictor of malignancy. Some have demonstrated higher SUV in malignant lesions [10,25], whereas others found minimal or no significant differences between benign and malignant lesions [26,27]. SUV does not consistently correlate with tumor differentiation [28].

In larger datasets, Bosgrud and Karantanis reported no significant difference between malignant (SUV ~6.4) and benign lesions (SUV ~5.6) [29]. In contrast, Choi and Lee reported higher SUV in malignant lesions (10.7 vs. 6.7) [30].

Median SUVs in malignant lesions are generally modest, including 6.3 reported in papillary thyroid carcinoma [31]. Abdulrezzak et al. reported SUVmax values of 5.3 in malignant versus 2.2 in benign lesions (*p* < 0.001) [4].

The higher mean SUV observed in malignant lesions in our cohort may reflect selection bias, as lesions with higher metabolic activity were more likely to undergo further evaluation and biopsy.

Overall, SUVs alone should not be used to distinguish benign from malignant thyroid incidentalomas. However, higher SUVs may warrant closer clinical attention and further diagnostic workup.

#### Bias and Limitation

First, selection bias may have been introduced due to the inclusion criteria and the single-center design, which may have resulted in a study population that is not fully representative of the broader patient population. Certain demographic or clinical subgroups could therefore be overrepresented or underrepresented due to referral patterns and institutional practices.

Second, information bias is possible due to reliance on existing medical records, which were not originally collected for research purposes. These records may be incomplete, inconsistently documented, or contain inaccuracies, potentially affecting the quality and reliability of the extracted data.

In addition, verification bias is present, as only a small number of incidental thyroid findings underwent cytological or histopathological confirmation. Accordingly, malignancy rates observed among biopsied lesions cannot be generalized to all PET/CT-detected thyroid incidentalomas.

Furthermore, follow-up was incomplete for a substantial proportion of patients with thyroid incidentalomas, which may have further influenced outcome ascertainment and introduced additional potential bias. This, together with the limited number of cytology- or histology-confirmed cases, further restricts the robustness of diagnostic classification and outcome assessment. The retrospective nature of the study also limits control over confounding variables and the uniformity of follow-up, contributing to residual bias.

Although standardized data extraction procedures were implemented in an attempt to reduce variability and improve data consistency, these inherent limitations cannot be fully eliminated. Therefore, the potential impact of these biases should be carefully considered when interpreting the study findings.

## 5. Conclusions

Incidental focal thyroid uptake on PET/CT was identified in approximately 3% of oncologic patients and appeared numerically more frequent among patients with breast cancer than among patients with other malignancies, although this difference was not statistically significant. A substantial proportion of patients with incidental thyroid findings were not referred for further evaluation, highlighting a potential gap in clinical management.

Among the subset of patients who underwent specialist assessment and FNA, papillary thyroid carcinoma was frequently identified. However, because only a selected subgroup of patients underwent further diagnostic workup, the observed malignancy rates are subject to verification/workup bias and should not be considered representative of all incidental thyroid lesions detected in the cohort.

Higher SUVs were associated with malignant findings in the biopsied subset, but the small sample size and selection bias limit the generalizability of this observation. Our results support increased awareness of incidental thyroid lesions detected on PET/CT and emphasize the importance of appropriate clinical evaluation, particularly in breast cancer patients, while larger prospective studies with systematic follow-up are needed to determine the true prevalence and risk of malignancy of these lesions.

## Figures and Tables

**Table 1:** Referral rate of thyroid incidentalomas to head and neck specialists.

	Not Referred to Head and Neck (*n* = 20)	Referred to Head and Neck (*n* = 20)	*p*
Age, y. (Me, 95% CI)	68.6, 63.4–74.0	68.6, 64.1–73.1	0.998
Ethnicity, Jewish (n, %)	12, 63.2	18, 85.7	0.148
SUV max, Dimensionless (Me, 95% CI)	5.8, 4.3–8.7	7.7, 5.5–15.0	0.117
Advanced disease (n, %)	9, 47.4	5, 23.8	0.119
Gender, Female (n, %)	15, 78.9	16, 76.2	0.835

Me—median, CI—confidence interval.

**Table 2:** Patient data: thyroid incidentaloma referred to head and neck surgeon.

Patient No.	Referred to E.N.T.	Age	Ethnicity	SUV	Status of Breast Cancer	Cytology	Final Treatment
1	+	39	Jewish	6.3	Non advanced	PTC	Hemithyroidectomy
2	+	66	Jewish	7.6	Non advanced	PTC	Total thyroidectomy + neck dissection
3	+	64	Jewish	4.3	Non advanced	Normal follicular cells	Hemithyroidectomy for goiter
4	+	79	Jewish	3	Non advanced	−	−
5	+	84	Jewish	16	advanced	PTC	metastatic breast cancer, PTC not treated
6	+	61	Arab	8	Non advanced	−	−
7	+	73	Jewish	18	advanced	PTC	Hemithyroidectomy
8	+	78	Arab	7.8	advanced	−	−
9	−	75	Jewish	9	advanced	−	−
10	−	64	Jewish	4.2	Non advanced	−	−
11	−	51	Jewish	10.3	advanced	−	−
12	−	36	Arab	17	Non advanced	−	−
13	−	79	Jewish	5	Non advanced	−	−
14	−	74	Jewish	4.2	Non advanced	−	−
15	−	73	Jewish	4.3	Non advanced	−	−
16	−	69	Jewish	3.8	advanced	−	−

## Data Availability

The datasets generated and/or analyzed during the current study are not publicly available due to patient privacy and institutional regulations but are available from the corresponding author upon reasonable request.

## Abbreviations

The following abbreviations are used in this manuscript:

SUV	Standardized Uptake Value
PTC	Papillary Thyroid Carcinoma
FNA	Fine-Needle Aspiration
CI	Confidence Interval
PET/CT	Positron Emission Tomography/Computed Tomography
